# A Carcinoid Tumor in the Urinary Bladder with Uncommon Clinicopathological Presentation

**Published:** 2017-07-01

**Authors:** Krishnendu Mondal, Rupali Mandal

**Affiliations:** 1 *Dept. of Pathology, Sonoscan Healthcare, Malda, India*

**Keywords:** Carcinoid Tumor, Chromogranin A Cystourethroscopy, Immunohistochemistry, Urinary Bladder

## Abstract

**Background:**

Carcinoid tumors usually originate from the enterochromaffin cells located in gastrointestinal tract and bronchopulmonary system. They may rarely arise in the urinary bladder, where this can be eventually miscued as any other commoner bladder neoplasms. The current study was conducted to connote an uncommon clinicopathological presentation by a carcinoid tumor in the urinary bladder.

**Case:**

A 52-year-old male, who initially experienced obstructive urinary symptoms, underwent cystourethroscopy to remove a tumor in the urinary bladder. The tumor exhibited insular, trabecular, and organoid architecture on histology without any necrosis or mitosis, stained positively with chromogranin A, and thereby, confirmed the diagnosis of a pure carcinoid tumor.

**Conclusion:**

Carcinoid tumors rarely arise in the urinary bladder and other genitourinary organs. But, several other and relatively more common bladder neoplasms may often deceptively simulate it. This dilemma could be resolved easily with the application of proper immunohistochemistry (IHC) in neuroendocrine tumors.

## Introduction

Carcinoid tumors rarely develop in the urinary bladder. Several earlier reports are recently derecognized by a number of scrutinizing literature reviews. Thus, collectively the number of bladder carcinoids identified so far barely amount to 2 dozen (1,2). Males between 26 to75 years are predominantly affected. They present haematuria with rarely urinary obstruction (3). The tumors are around 3 to12 mm in diameter. They generally exhibit a glandular or cribriform architecture; occasionally, trabecular or organoid morphology may also coexist (1). Owing to the extreme rarity, a small cell carcinoma or solid urothelial carcinoma is often misinterpreted as carcinoid in the bladder. Such confusion is promptly settled by silver stains, IHC, etc. (3). The current report narrates the clinical, cystoscopic, histopathological, and immunohistochemical properties of a pure carcinoid tumor in the urinary bladder.

## Case Report

A 52-year-old male underwent urological evaluation after initially complaining about obstructive micturition symptoms such as the sense of incomplete bladder emptying, frequent urination, and nocturia. Any relevant medical, occupational, or addiction history was negative. His morning urine sample appeared ‘smoky’, which detected hematuria on routine examination. His prostate was smooth-surfaced and unremarkable, based on the clinico-radiological examinations. Subsequent abdomino-pelvic ultrasonography also failed to reveal any contextual pathology.

**Fig 1:**
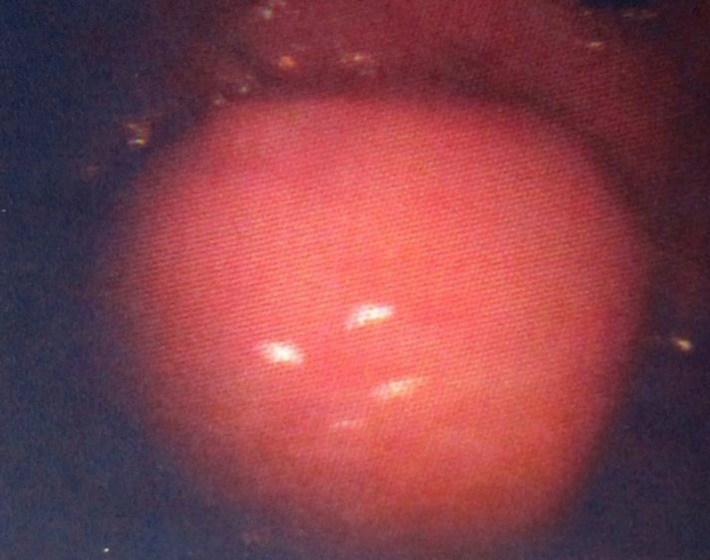
Bladder carcinoid: Polypoid nodule with smooth pink surface, based on the cystoscopical examination

**Fig 2:**
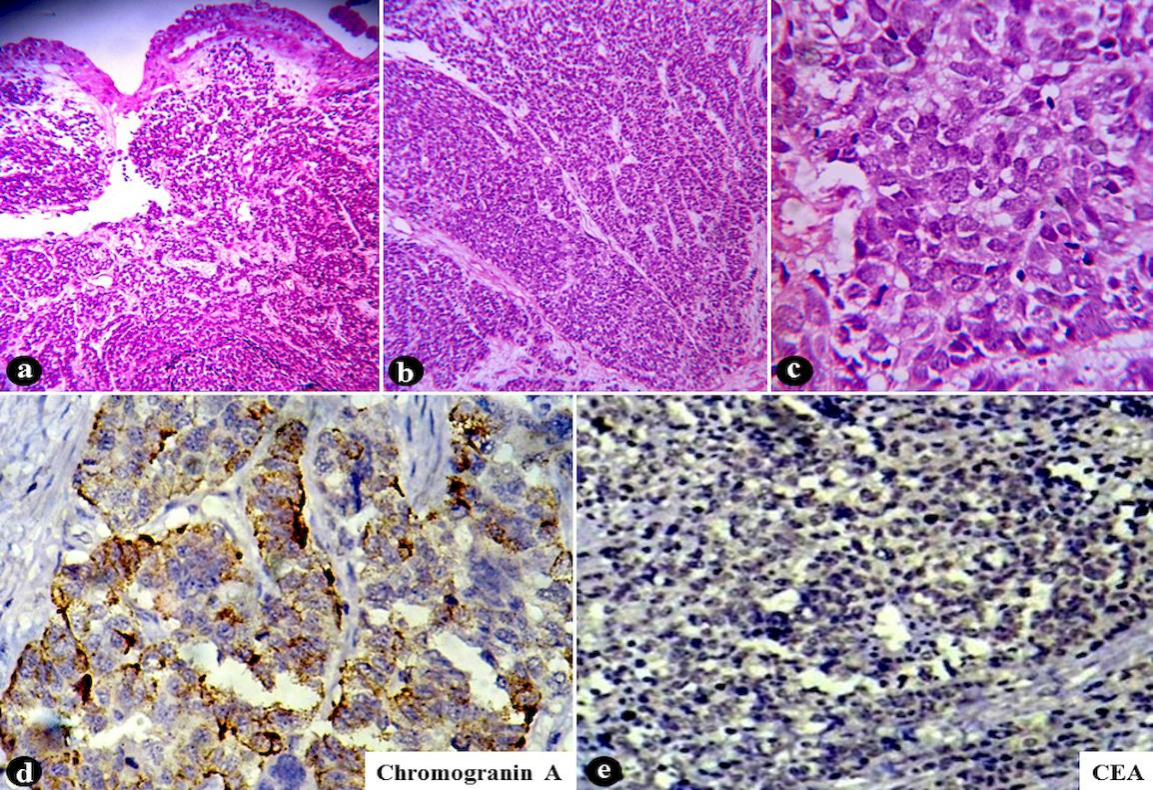
Bladder carcinoid: Normal intact urothelium overlying the branching trabeculae (a), and insular fronds (b) of carcinoid cells [H&E stain, 40x], with rounded nuclei and ‘salt-and-pepper’ chromatin (c) based on the histopathological examination [H&E stain, 400x]; tumor cells stain positive for chromogranin A (d) [400x], and negative for CEA (e) [100x].

On cystourethroscopy, a 7-mm sessile polypoid nodule, bearing smooth and glistening pink surface, was identified at the bladder neck adjacent to urethral ostium (Figure 1). Transurethral resection of the tumor was performed. Microscopically, the urothelium maintained its normal morphology. The tumor produced a circumscribed, but expansile growth within the lamina propria. It was composed of uniform round-to-ovoid neoplastic cells in thick anastomosing trabeculae and insular structures delineated by thin-walled blood vessels. The neoplastic cells contained solitary rounded nuclei with finely granular stippled chromatin imparting the classic ‘salt-and-pepper’ quality, inconspicuous nucleoli, and abundant granular eosinophilic cytoplasm. Mitosis or necrosis was inapparent. Immunohistochemical staining demonstrated diffuse strong cytoplasmic granular positivity for chromogranin A. Antibodies against carcinoembryonic antigen (CEA) were non-reactive (Figure 2). The tumor was then unanimously diagnosed as carcinoid tumor of the urinary bladder.

Postoperatively, the patient was gradually relieved symptomatically. Cystoscopy was performed at 6- and 12-month intervals and no symptom of recurrence was observed. Follow-up abdominal computed tomography (CT) was innocuous as well.

## Discussion

Neuroendocrine tumors (NETs) represent 1.7% of all vesical neoplasms (4). Small cell carcinoma is its vast predominant constituent. Other infrequent bladder NETs include paraganglioma, carcinoid, primitive neuroectodermal tumor, neuroblastoma, and large cell neuroendocrine carcinoma. Although extremely rare, carcinoid still ranks the 3rd commonest among the NETs (3,5). Several earlier reports on carcinoids were misdiagnoses under critical literature reviews by Martignoni et al. (1) and Chen et al. (2). Implicit intermingling and/or juxtaposition of carcinoid with small cell carcinoma lead to such misinterpretations in the cases (1). 

Carcinoid tumors primarily arise in the alimentary and lower respiratory tracts; whereas their genitourinary incidence in the testis, prostate, kidney, urethra, or urinary bladder is only encountered incidentally (6). Vesical carcinoid has a wide age distribution from 26 to 75 years. Males are affected twice more frequently than females (3,5). Almost all of the cases are located in proximity to the trigone and neck region. Morphologically, the tumors are subepithelial in location, confined to the lamina propria, sometimes accompanied by cystitis cystica or glandularis (2). Patients generally present hematuria, and less often the voiding disturbances. None of the patients yet reported carcinoid syndrome (3,5). In this respect, Mascolo et al. (7) immunohistochemically recognized a calcitonin-producing tumor, though it had the least influence on the overall symptomatology. The discussed patient was not different from the previous literatures. The tumor nodule produced micturition difficulties. Hematuria was not grossly obvious, but it was detected on routine urinary microscopy. 

Architecturally, carcinoid tumors are arranged in glandular, insular, trabecular, or composite patterns. Glandular arrangement is by far the most common characteristic of bladder carcinoids (1). Rare tumors containing carcinoid, small cell carcinoma, and adenocarcinoma components are also described in the bladder (5). The histogenesis of bladder carcinoids is debated as: Direct tumorigenesis from neuroendocrine cells, epithelial metaplasia, multipotential stem cell derivation, etc. Whatever may be the etiopathogenesis, these carcinoid cells generally express argyrophilia, with some extent of argentaffinity (6). Their neuroendocrine differentiation is best elicited by the immunohistochemical markers such as chromogranin, synaptophysin, and neuron-specific enolase (4,5). Similarly, the observed tumor exhibited an insular-trabecular composite architecture without the signature glandular pattern. Immunohistochemical positivity for chromogranin A confirmed its neuroendocrine derivation.

Due to its extreme rarity in the urinary bladder, carcinoid tumor is actually a diagnosis of exclusion. Small cell carcinoma with carcinoid-like areas and urothelial carcinoma with nested growth or focal neuroendocrine differentiation often closely simulate a carcinoid. Careful categorization of cytologic features, absence of significant necrosis/mitosis, strong and diffuse immunoreactivity for neuroendocrine marker(s), and negativity for epithelial antigens isolate the proper diagnosis (2,3). Likewise, in the current report all those differentials were stepwise excluded, on the basis of histopathology followed by immunohistochemistry, to clinch the diagnosis of carcinoid tumor.

Bladder carcinoids are successfully cured by transurethral removal. But rare lesions do recur or metastasize (3). The current report patient did not experience any relapse during his 1-year follow-up, recapitulating similar observations from the already-published literature.

In summary, carcinoid tumor, a rare urinary bladder neoplasm, typically presents with hematuria with or without voiding difficulties. It usually exhibits a glandular architecture, but uncommonly trabecular or insular arrangement is also observed. Diagnostic dilemma whenever arises could effectively be settled by proper immunohistochemical staining. Cystoscopic removal of mass is the treatment of choice. Follow-up with abdomino-pelvic CT and cystourethroscopy offers the best surveillance approach in such patients.
